# A database for risk assessment and comparative genomic analysis of foodborne *Vibrio parahaemolyticus* in China

**DOI:** 10.1038/s41597-020-00671-3

**Published:** 2020-10-02

**Authors:** Rui Pang, Yanping Li, Moutong Chen, Haiyan Zeng, Tao Lei, Junhui Zhang, Yu Ding, Juan Wang, Shi Wu, Qinghua Ye, Jumei Zhang, Qingping Wu

**Affiliations:** 1 Guangdong Provincial Key Laboratory of Microbial Safety and Health, State Key Laboratory of Applied Microbiology Southern China, Guangdong Institute of Microbiology, Guangdong Academy of Sciences, Guangzhou, 510070 China; 2 Department of Food Science and Technology, Jinan University, Guangzhou, 510000 China; 3 College of Food Science, South China Agricultural University, Guangzhou, 510642 China

**Keywords:** Pathogens, Infection, Microbial ecology

## Abstract

*Vibrio parahaemolyticus* is a major foodborne pathogen worldwide. The increasing number of cases of *V. parahaemolyticus* infections in China indicates an urgent need to evaluate the prevalence and genetic diversity of this pathogenic bacterium. In this paper, we introduce the Foodborne *Vibrio parahaemolyticus* genome database (FVPGD), the first scientific database of foodborne *V. parahaemolyticus* distribution and genomic data in China, based on our previous investigations of *V. parahaemolyticus* contamination in different kinds of food samples across China from 2011 to 2016. The dataset includes records of 2,499 food samples and 643 *V. parahaemolyticus* strains from supermarkets and marketplaces distributed over 39 cities in China; 268 whole-genome sequences have been deposited in this database. A spatial view on the risk situations *of V. parahaemolyticus* contamination in different food types is provided. Additionally, the database provides a functional interface of sequence BLAST, core genome multilocus sequence typing, and phylogenetic analysis. The database will become a powerful tool for risk assessment and outbreak investigations of foodborne pathogens in China.

## Abstract (summary)

**Table:** 

Measurement(s)	Microbial Contamination • sequence_assembly • DNA
Technology Type(s)	the most probable number method • sequence assembly process • DNA sequencing
Sample Characteristic - Organism	Vibrio parahaemolyticus
Sample Characteristic - Location	China

Machine-accessible metadata file describing the reported data: 10.6084/m9.figshare.12629891

## Background & Summary

*Vibrio parahaemolyticus* is a halophilic, gram-negative bacterium that is commonly found in estuarine and marine environments worldwide. This microorganism is recognized as one of the most prevalent foodborne pathogens and typically causes acute gastroenteritis in humans^1^. This bacterium preferentially grows in warm and low-salinity marine water and sometimes colonizes aquatic hosts such as mollusks, shrimp, and fish^2^. Most people are infected by eating raw or undercooked seafood^3,4^. *V. parahaemolyticus* can also cause necrotizing fasciitis through wound infection^5^. *V. parahaemolyticus* outbreaks have been reported in many countries such as Bangladesh^6^, Italy^7^, Japan^8^, Brazil^9^, and the USA^10^.

China, a vast country with the largest population in the world, has a high rate of seafood consumption. As a result of this, *V. parahaemolyticus* has been the leading cause of foodborne outbreaks and cases of infectious diarrhea in China, especially in its coastal regions^11,12^. In the city of Sanya, China, alone, there were 29 outbreaks caused by *V. parahaemolyticus* resulting in 499 cases from 2010 to 2016, accounting for about half of all foodborne microbial infections^13^. To characterize the prevalence and genetic diversity of *V. parahaemolyticus* in foods in China, we collected food samples from all over China from July 2011 to July 2016 and tested for contamination by *V. parahaemolyticus*^14–17^. Remarkably, *V. parahaemolyticus* contamination was not only found with a high rate in aquatic products but also in ready-to-eat (RTE) foods in most cities in China^17^. As all previous studies were published independently and focused on a coarse food type-level, there is a need to integrate this information, with more complete records that include the genetic background of foodborne *V. parahaemolyticus* strains at the whole-genome level.

Here, we have constructed the Foodborne *Vibrio parahaemolyticus* Genome Database (FVPGD), a database comprising 2,499 records of aquatic products and RTE foods collected from 39 cites in China, from which, a total of 643 *V. parahaemolyticus* strains were isolated. The whole-genome sequences of most strains were obtained using a next-generation sequencing strategy and deposited in this database. A core genome multilocus sequence typing (cgMLST) scheme was provided to analyze the epidemiology of *V. parahaemolyticus* in China according to the genomic information. This database demonstrates the risk level of *V. parahaemolyticus* contamination in different kinds of food samples collected from all over China and provides a series of high-quality genomes for investigating the genetic relationships of foodborne *V. parahaemolyticus* strains from multiple temporal-spatial niches. Moreover, this database will facilitate the risk assessment of foodborne *V. parahaemolyticus* in China and contamination tracing of *V. parahaemolyticus* infections in the future.

## Methods

### Data collection

**Fig. 1:**
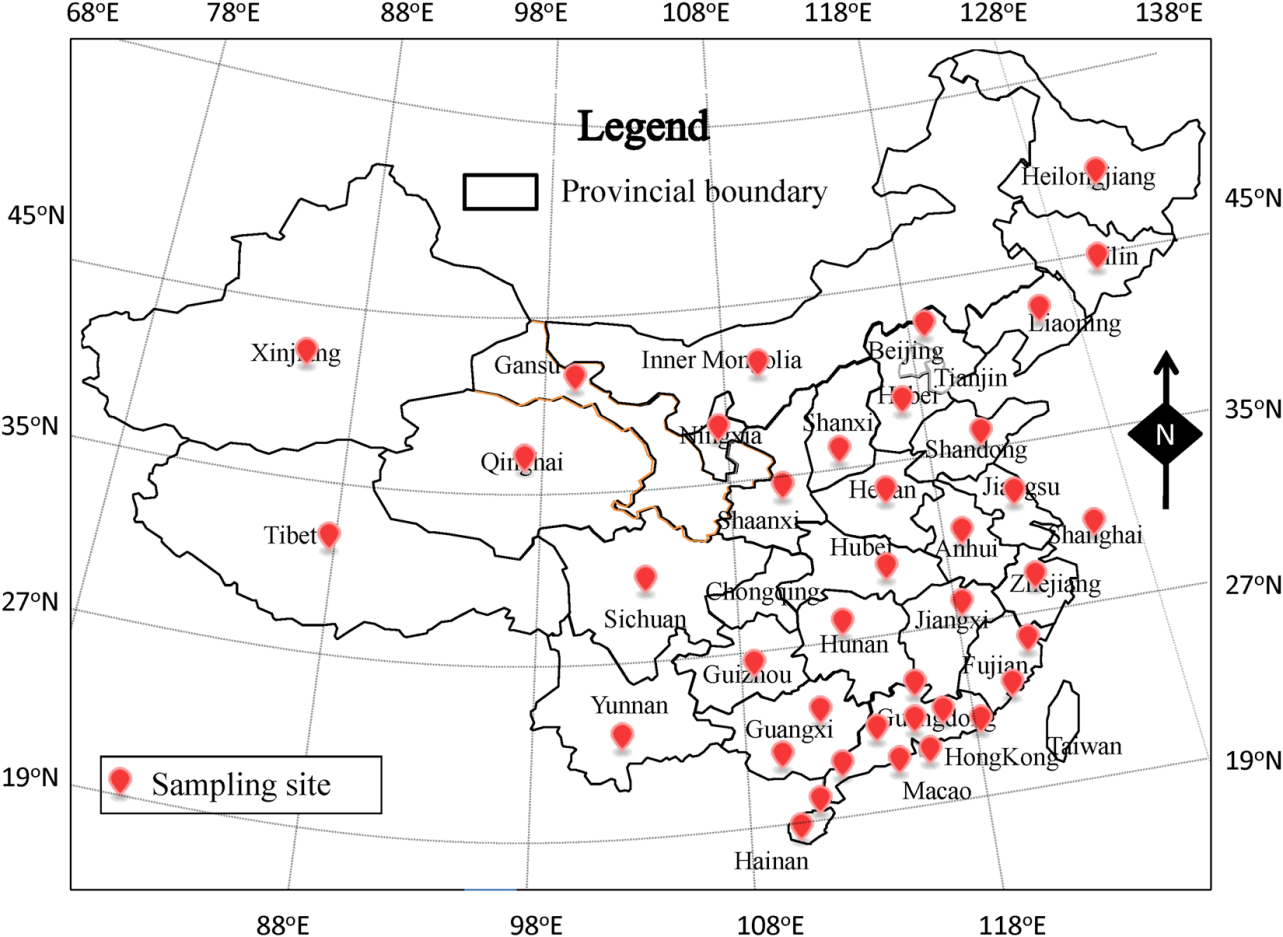
Food sampling sites in China for this study.

From July 2011 to July 2016, a total of 2,499 food samples, including 1,640 aquatic products and 859 RTE foods, were collected from supermarkets and marketplaces from most provincial capitals in China (Fig. 1). A total of 643 *V. parahaemolyticus* strains were isolated from 574 positive samples according to the GB 4789.7-2013 food microbiological examination of *V. parahaemolyticus* (National Food Safety Standards of China) and the most probable number method^14–17^. They were further identified by the analysis of oxidase activity, Gram staining, 3.5% NaCl triple sugar iron tests, halophilism tests, and API 20E diagnostic strips (Biomerieux Company, France). For epidemiological analysis, we also sampled 16 clinical *V. parahaemolyticus* strains isolated from patients in Guangdong, China, from 2011 to 2018.

### Genome sequencing

*V. parahaemolyticus* strains were grown overnight at 37 °C in tryptic soytone broth medium (HuanKai Microbial, Guangzhou, China). Genomic DNA was extracted from *V. parahaemolyticus* strains using a genomic DNA extraction kit (Magen Biotech, Guangzhou, China) according to the manufacturer’s instructions. DNA samples were fragmented into 400-bp fragments by a Covaris M200 sonicator and used to generate sequencing libraries. Whole genomes were sequenced on the Life Ion S5 platform or Illumina Miseq/Nextseq 550 platform with an average coverage of 100× as previously described^18^. Raw reads were subjected to adapter clipping and quality filtering, and the obtained clean reads were assembled de novo by SPAdes v3.6.2 software^19^. All sequence data were checked for contamination using the Contamination Screen module of the submission system of NCBI, and then the sequences that needed to be trimmed and/or excluded were removed from the assemblies accordingly.

### Definition of cgMLST scheme

For the cgMLST scheme construction, the complete genome sequences of six reference *V. parahaemolyticus* strains [RIMD2210633 (GenBank accession no. GCA_000196095.1), ATCC17802 (GenBank accession no. GCA_001558495.1), BB22OP (GenBank accession no. GCA_000328405.1), CDC_K4557 (GenBank accession no. GCA_000430425.1), FDA_R31 (GenBank accession no. GCA_000430405.1), and CHN25 (GenBank accession no. GCA_001700835.1)] were downloaded from NCBI and added to our database. The protein-coding genes of all genome sequences were annotated using Prokka v1.11^20^. The output of Prokka was used to create the pan-genome of *V. parahaemolyticus* with Roary v3.11.2^21^. The core genes were then determined for each isolate with a BLASTN identity cutoff of 85% and with a cutoff of their presence in more than 99% of the strains, as previously described^18^. To ensure the computing speed of further analysis, core genes that lacked functional annotation or were annotated as hypothetical proteins were excluded. Genes that were present in more than one copy in any of the reference genomes were also removed. Finally, 672 core genes were selected, and their reference sequences (the same as that of strain RIMD2210633) were used for the cgMLST scheme. A genome-wide gene-by-gene cgMLST comparison was performed with every genome queried against the reference sequences, with the BLASTN threshold of identity >85% and coverage >90%. Alleles for each gene were assigned automatically by a local script. The combination of all alleles in each strain formed an allelic profile and the missing core genes were excluded from this profile.

### Phylogenetic analysis of *V. parahaemolyticus* strains

A phylogenetic analysis of *V. parahaemolyticus* strains was performed using the concatenated alignment of 672 core genes. Missing core genes in each genome were ignored for the estimation of phylogenetic relationships as previously reported^22^. Each nucleotide sequence was aligned with MAFFT v7.310^23^, and the concatenated alignment was used to infer an approximate maximum likelihood phylogeny by FastTree v2.1.10 with default parameters (FastTree -gtr -nt alignment_file > tree_file)^24^.

### BLAST search

The NCBI BLAST + v2.7.1 software was integrated as a web-service in our database. The genome sequences, protein sequences, and protein-coding sequences from each *V. parahaemolyticus* strain were used as the BLAST database.

## Data Records

The dataset consists of two groups of data. The first group has the full information of all collected food samples, including their spatial and temporal distributions, sample types (e.g., fish, shrimp, and squid), detail of collection (supermarket or marketplace), and condition of contamination. The second group describes the background of all isolated *V. parahaemolyticus* strains, including their spatial and temporal distributions, isolated sources, serotypes, and sequencing information. These two data groups are connected through a unique ID for the food samples and have been uploaded to the figshare repository^25^. Additionally, genome sequences and genomic annotations were linked to *V. parahaemolyticus* strains through a unique ID for each strain and the corresponding files have been deposited in figshare^26^. All sequencing reads have been deposited in the NCBI Sequence Read Archive database under the SRA study accession SRP253458^27^.

The dataset is managed locally in a shared database and is accessible publicly at http://210.77.86.67/VP.html. The online dataset is updated when new genomes of *V. parahaemolyticus* strains are sequenced and when there are new collections of food samples.

## Technical Validation

**Fig. 2:**
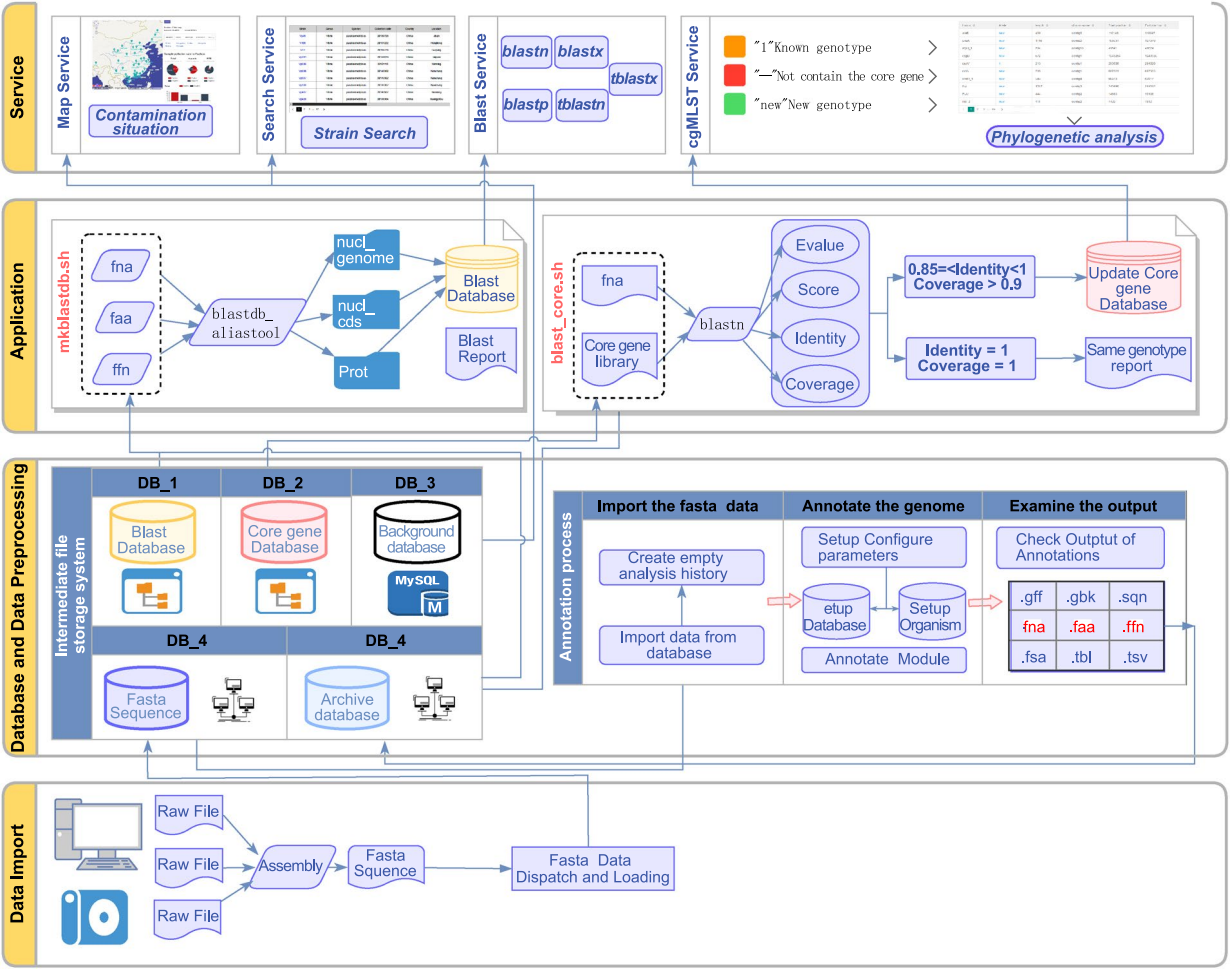
Schematic overview of the structure and data schedule of the FVPGD.

Information on the food samples and *V. parahaemolyticus* strains has been deposited as primary data in the FVPGD and is managed locally to ensure its validity and timeliness. The genome sequencing data of *V. parahaemolyticus* strains were processed following the schematic overview shown in Fig. 2. All genome sequences were checked for their GC content (within 44%~46%), contig number (≤300), and contig N50 (>50 kb) (Supplementary Table 1) before importing to the database. The processed data were transferred to the web service for browsing and comparison.

**Fig. 3:**
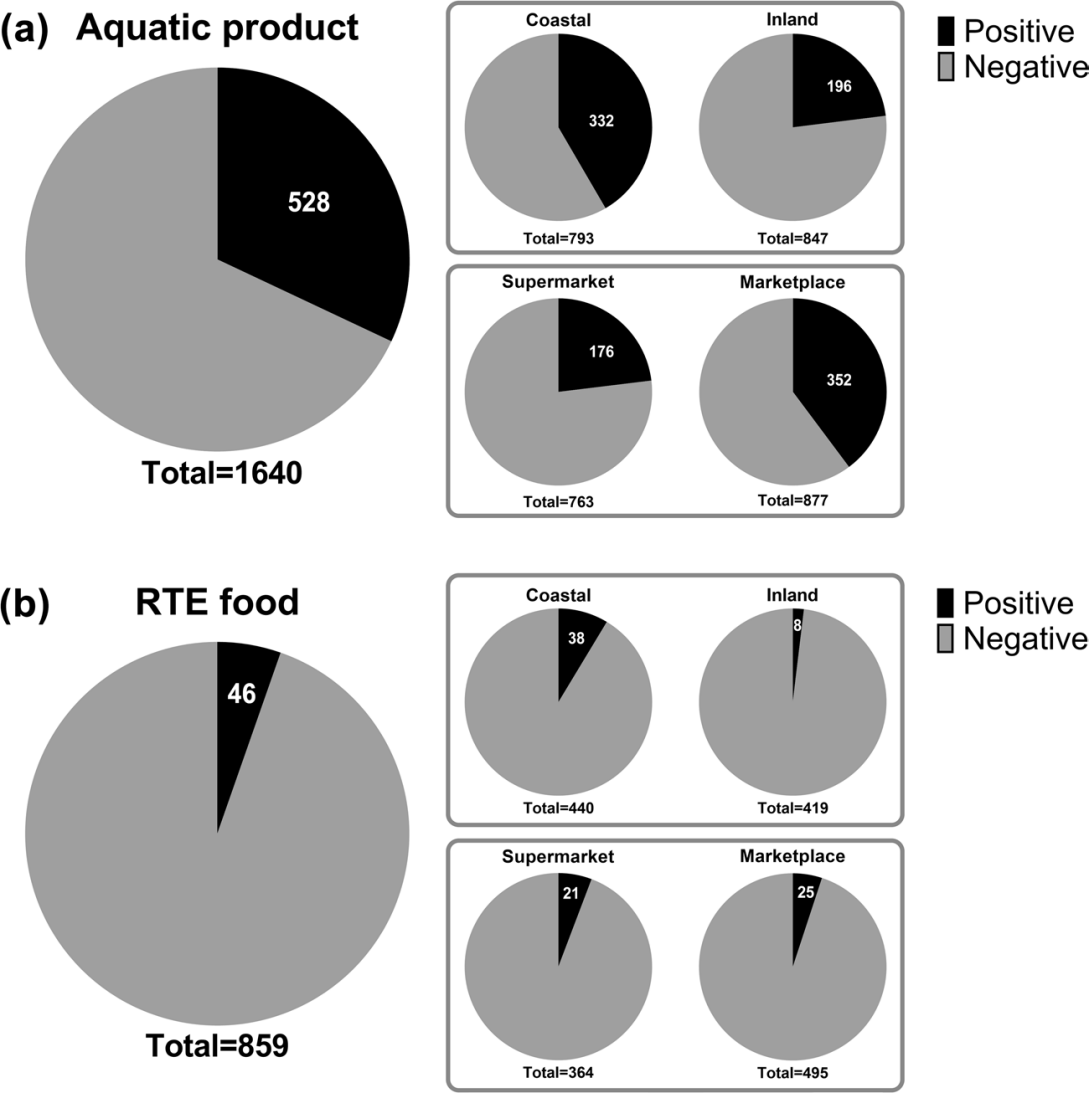
Statistics of *V. parahaemolyticus* contamination situations in China. (**a**) Contamination rate of *V. parahaemolyticus* in aquatic products. (**b**) Contamination rate of *V. parahaemolyticus* in RTE foods.

For aquatic products, the total rate of *V. parahaemolyticus* contamination in China was 32.20% (Fig. 3a). However, there was a great difference between coastal provinces and inland provinces. The contamination rate in coastal provinces (41.87%) was higher than that in inland provinces (23.14%), which was in accordance with the high level of foodborne *V. parahaemolyticus* infections in the coastal cities in China^11,28^. Additionally, aquatic products collected from marketplaces showed a much higher contamination rate of *V. parahaemolyticus* (40.14%) than those collected from supermarkets (23.07%), reflecting that the standardized procurement conditions in supermarkets might significantly reduce the risk of *V. parahaemolyticus* infection. The total rate of *V. parahaemolyticus* contamination in RTE foods from China was 5.36% (Fig. 3b). Although lower than that of aquatic products, its risk should not be ignored because RTE foods do not require heat treatment or other forms of curing before eating^17^. The *V. parahaemolyticus* contamination rate of RTE foods was much higher in coastal provinces (8.64%) than in inland provinces (1.91%), which is similar to that observed in aquatic products. However, there was no difference between RTE foods collected from supermarkets (5.77%) and marketplaces (5.05%). This might be an indication of *V. parahaemolyticus* persistence in RTE foods^18^.

**Fig. 4:**
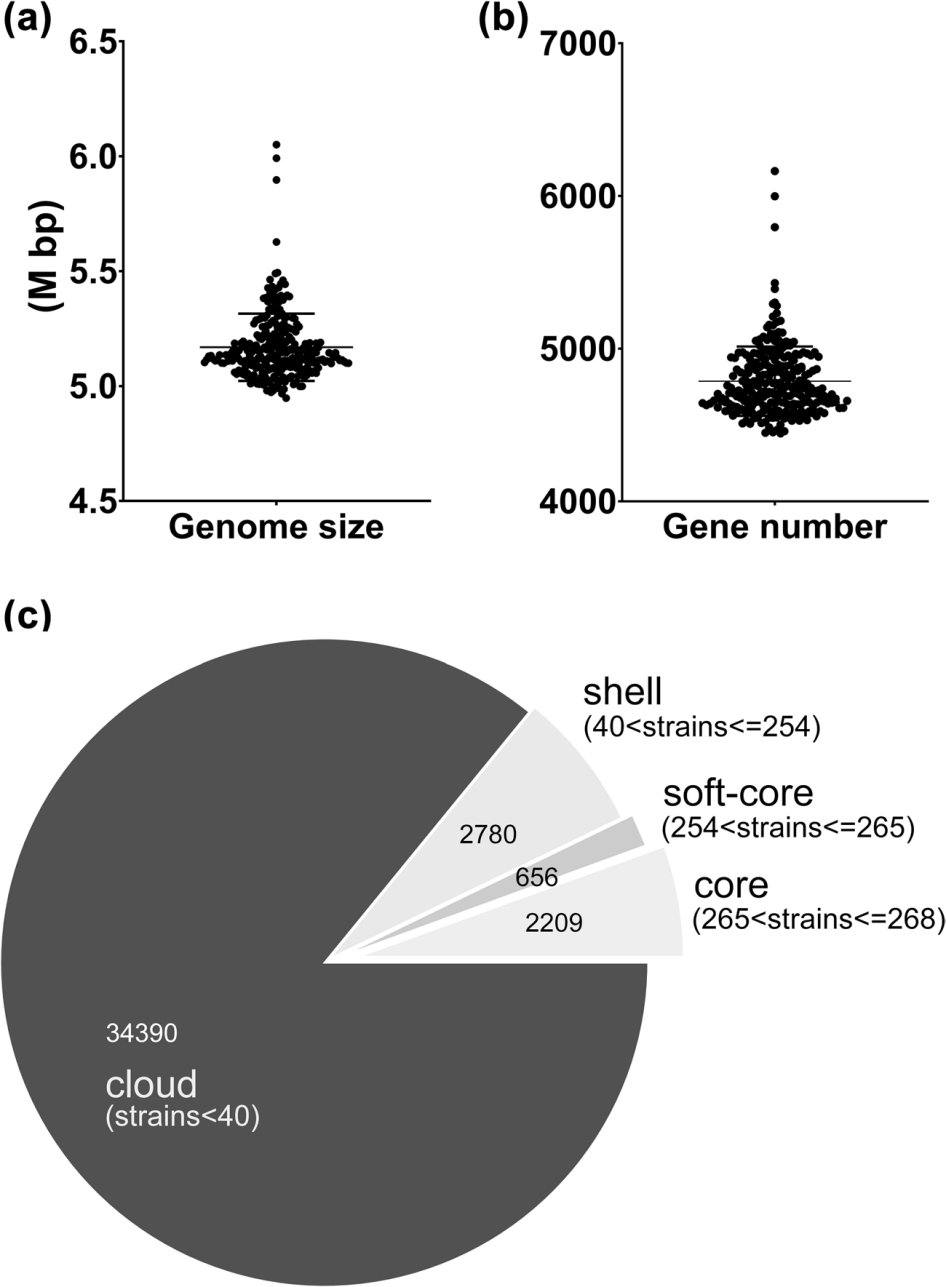
Overview of the genomic data deposited in the FVPGD. (**a**) Genome sizes of *V. parahaemolyticus* strains. (**b**) Protein-coding number of *V. parahaemolyticus* strains. (**c**) Pan-genome distribution of *V. parahaemolyticus* strains.

Up to now, the FVPGD has recorded the genome sequences of 268 *V. parahaemolyticus* strains, including 6 reference strains, 16 clinical strains, and 246 food-sourced strains. Among them, the sequences of 39 food-sourced strains have been previously reported^18^. The genome sizes of these strains ranged from 4.95 to 6.05 M bp (Fig. 4a). These genomes contained an average protein-coding gene number of 4787 (Fig. 4b). Additionally, the pan-genome of these strains consisted of 40,035 protein-coding genes, of which, 2,209 core genes were identified (Fig. 4c). It has been reported that *V. parahaemolyticus* harbors an open pan-genome, the gene content of which will increase as more strains are analyzed^29,30^. The pan-genome size obtained in this analysis was rather large. In contrast, the content of core genes remains stable and is very close to the results of previous analyses^29,31^. Further analysis revealed that 672 core genes were annotated with definitive functions, and these genes were used for the cgMLST scheme of this database according to a previous report^31^. Although they differed in topological structure, the phylogenetic trees generated from this gene set and full core genes were identical in the clustering of homologous strains (Supplementary Fig. 1), indicating that the selected gene set is sufficient to distinguish related and unrelated strains in a genome-wide resolution.

## Usage Notes

**Fig. 5:**
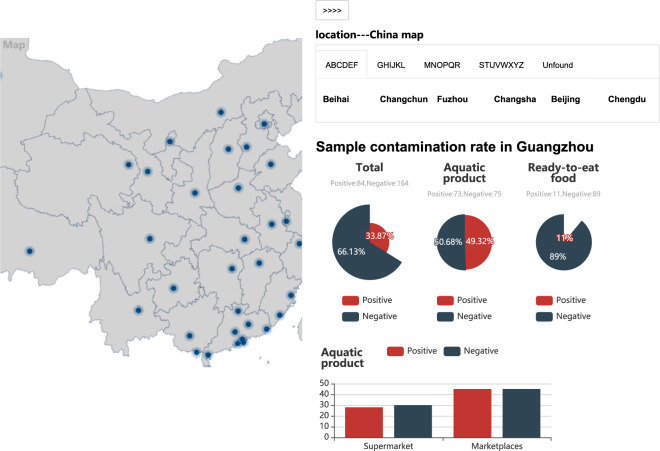
Spatial view on the risk situations of *V. parahaemolyticus* contamination in the FVPGD.

*V. parahaemolyticus* strains included in the FVPGD can be directly retrieved according to their characteristics, such as location, source, and gene name. Furthermore, the information on *V. parahaemolyticus* strains can also be linked through a geographical map showing the risk situations of *V. parahaemolyticus* contamination in different cities in China (Fig. 5).

**Fig. 6:**
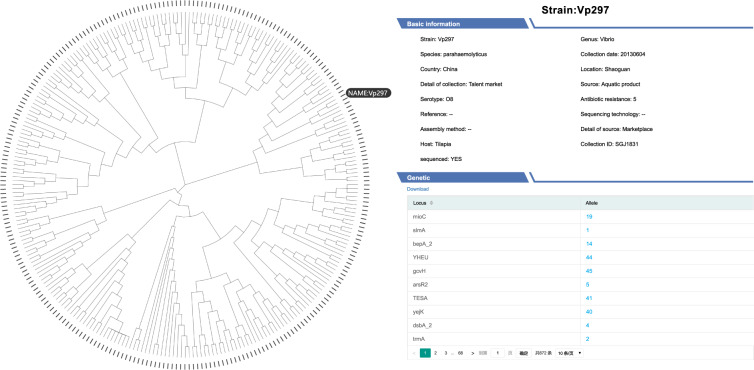
Phylogenetic tracing of *V. parahaemolyticus* strains in the FVPGD.

In the functional interface of cgMLST, users can upload the genome sequence of their *V. parahaemolyticus* strains. The sequence will be automatically BLASTed to the reference sequences of all core genes. If a core gene of a query sequence is identical to one in the sequences stored in the FVPGD, then the corresponding genotype number will be displayed; otherwise, the query core gene will be annotated as a new genotype. The users can compare their strains to all the strains in this database through a phylogenetic analysis based on the aligned sequences of all core genes. From the output phylogenetic tree, the input *V. parahaemolyticus* strain can be traced to the most homologous strain to determine its potential source (Fig. 6).

## Supplementary information

Supplementary Table 1

Supplementary Figure 1

## Data Availability

The versions of any software used and any specific variables or parameters used to process the current dataset have been detailed in the Methods section. The custom code produced during the validation of this dataset and the source code that underlies the interactive web interface (http://210.77.86.67/VP.html) have been shared on GitHub (https://github.com/pr839ok/FVPGD).
