# Supplementary material for: A database for risk assessment and comparative genomic analysis of foodborne Vibrio parahaemolyticus in China
Source: Sci Data. 2020 Oct 2;7:321. doi: 10.1038/s41597-020-00671-3 (PMC7532206; doi:10.1038/s41597-020-00671-3)
Supplement: Supplementary file 2 — Supplementary Figure 1 [file 41597_2020_671_MOESM2_ESM.pdf]

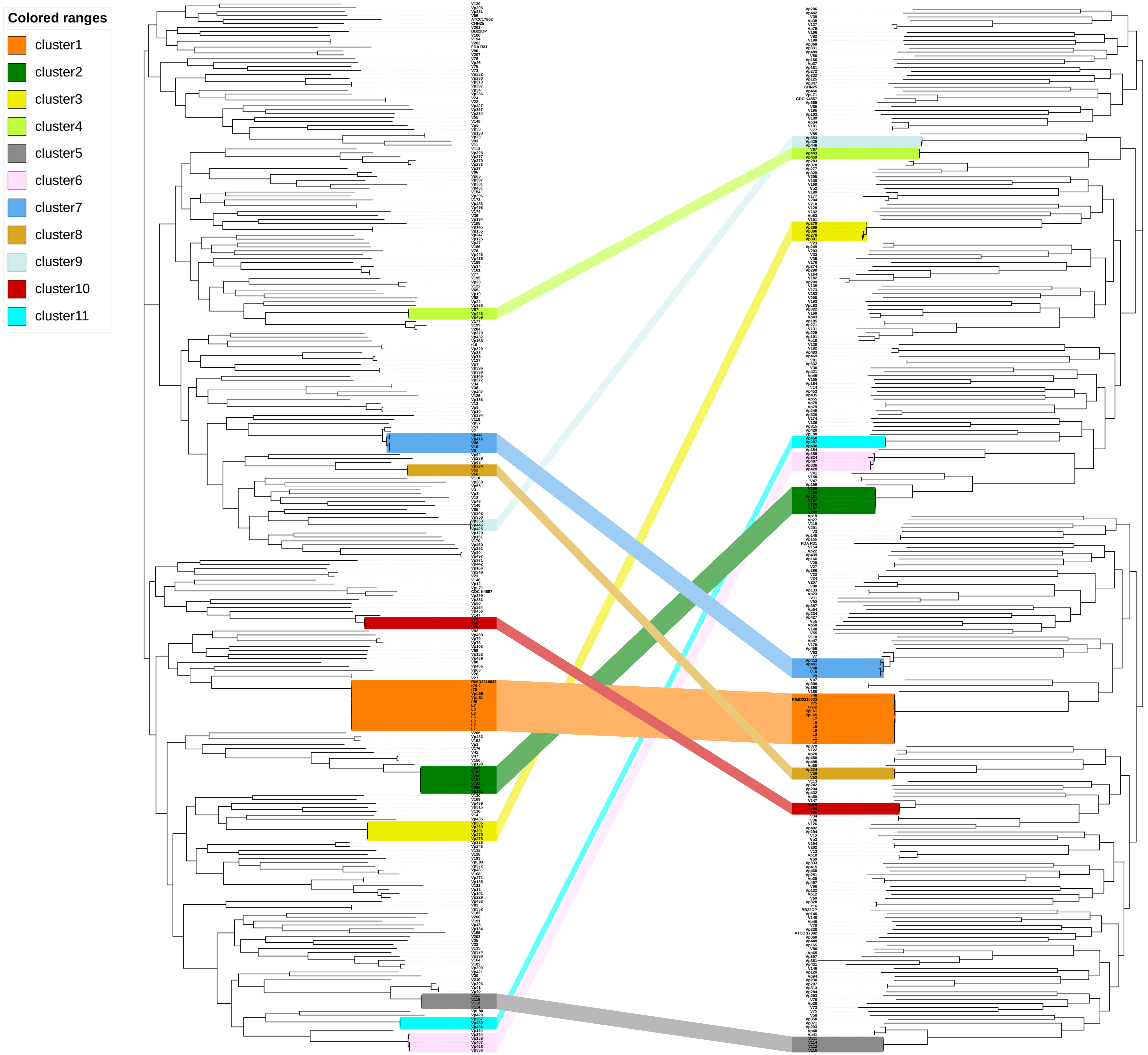
Colored ranges
cluster1
cluster2
cluster3
cluster4
cluster5
cluster6
cluster7
cluster8
cluster9
cluster10
cluster11
